# Preparation and Characterization of Sodium Caseinate-Coated Papers Based on Glycerol and Sorbitol Contents for Packaging Application

**DOI:** 10.3390/foods12050940

**Published:** 2023-02-22

**Authors:** Dowan Kim, Jihyeon Hwang

**Affiliations:** Department of Food Processing and Distribution, College of Life Science, Gangneung-Wonju National University, 7 Jukheon-gil, Gangneung 25457, Gangwon-do, Republic of Korea

**Keywords:** paper-based packaging, sodium caseinate, glycerol, sorbitol, food packaging, sustainability

## Abstract

Bio-based packaging materials are promising alternatives to petroleum-based plastics. Paper-based packaging materials are candidates for improving food sustainability; however, paper has poor gas and water vapor barrier properties. In this study, entirely bio-based sodium caseinate (CasNa)-coated papers with two plasticizers, glycerol (GY) and sorbitol (SO), were prepared. The morphological and chemical structure, burst strength, tensile strength, elongation at break, air permeability, surface properties, and thermal stability of the pristine CasNa-, CasNa/GY-, and CasNa/SO-coated papers were evaluated. The use of GY and SO strongly affected the tensile strength, elongation at break, and air barrier of the CasNa/GY- and CasNa/SO-coated paper. The air barrier and flexibility of the CasNa/GY-coated papers were higher than those of the CasNa/SO-coated papers. Compared to SO, GY better coated and penetrated the CasNa matrix, which positively affected the chemical and morphological structure of the coating layer and the interaction between the coating layer and paper. Overall, CasNa/GY was superior to the CasNa/SO coating. CasNa/GY-coated papers may be a good alternative for packaging materials in the food, medical, and electronic sectors, which would promote sustainability.

## 1. Introduction

Petroleum-based plastics such as polyethylene (PE), polypropylene (PP), and polyethylene terephthalate (PET) are made by linking hundreds of fossil oil-based monomers by chemical reactions [1]. They have excellent chemical stability, mechanical strength, transparency, and gas barrier properties, making petroleum-based plastics suitable for various packaging applications, such as food, medical devices, and electronic devices [1,2]. However, the global increase in the use of non-renewable petroleum plastics has negatively affected the environment. In response to environment issues, the European Union has published guidelines to ban single-use plastic products such as straws, food, and beverage containers [3].

Bio-based materials are promising alternatives to reduce the use of petroleum-based plastics for packaging [2,4,5,6,7]. Paper-based packaging materials are widely used in various foods, such as snacks, powdered foods, beverages, and frozen foods. Paper-based packaging materials have various advantages, such as ease of recyclability, degradability after use, and good mechanical properties [5,7]. However, paper has poor gas and water vapor barriers because of its porous and hydrophilic structure and lack of heat sealability [5]. Therefore, paper is usually coated and laminated with various plastics, such as PE and PP, to improve its properties. Unfortunately, plastic-coated and laminated paper lose their advantages as a sustainable material because of the difficulties in separating plastic from paper and their non-biodegradability [5,8].

Extensive research has been conducted on coated paper prepared using bio-based and renewable materials, such as zein, proteins, starch, nanocellulose, and polyvinyl alcohol, instead of plastics [5,6,7,8,9]. Coating is a process in which non-porous paper can be obtained by covering the porous structure and surface of cellulose fiber, improving its gas and water vapor barrier properties [8,9]. Sodium caseinate (CasNa) is a natural water-soluble polymer originating from the acid precipitation and neutralization of casein from milk and cheese production [2,4]. CasNa has relatively good film forming and coating abilities, making it an attractive candidate for preparing edible and biodegradable films. Furthermore, CasNa-based films exhibit good oxygen and aroma barrier properties due to intermolecular interactions, such as hydrogen bonding, which increase the intermolecular cohesion to form films [2,4,5,6]. However, these chemical and physical interactions cause CasNa films to shrink during the drying process at high temperatures and become brittle [6,10]. This hydrophilic structure negatively affects the physical properties of this film, making it a poor water barrier that restricts its various applications in food packaging [2,4,5,6]. 

To solve this problem, several researchers have reported the plasticizer effect using polyols such as GY, SO, polyethylene glycol (PEG), and stearic acid (SA). Plasticizers interrupt the intermolecular interactions of protein chains and induce the hydrogen bonds between protein chains and plasticizer, which improve the workability, flexibility, and brittleness of CasNa [5,11,12,13]. Colak et al., reported that GY-plasticized CasNa with non-brittleness was successfully prepared via solution casting and a brown extrusion process [11]. Rezvani et al., showed that hydrophobic SA strongly affects the drying process, mechanical strength, and water vapor permeability of CasNa films [12]. Siew et al., prepared CasNa/GY and CasNa/PEG edible films and concluded that the interaction and compatibility between the plasticizer and CasNa strongly influenced the viscosity of the coating solution and the physical properties of the film [13]. Khwaldia reported that the water vapor permeability of CasNa/paraffin wax-coated papers was less permeable to water vapor and weaker than that of only CasNa-coated paper [5]. Despite considerable research on the processing and physical properties of edible CasNa films depending on a plasticizer, only a few studies have reported the properties of CasNa-coated paper. 

Therefore, the present study aimed to prepare CasNa/GY-coated and CasNa/SO-coated papers using the solution blending method. In addition, their chemical and morphological structures were evaluated and compared depending on the plasticizer content to determine their suitability for various packaging applications.

## 2. Materials and Methods

### 2.1. Materials

The base paper (bleached kraft pulp, grammage: 80 g/m^2^, brightness 84%, and thickness: 95 µm) was purchased from OPI Co., Ltd. (Anseong, Republic of Korea). The casein sodium salt from bovine milk was purchased from Merck (Seoul, Republic of Korea). GY and SO were purchased from Duksan Pure Chemical Co., Ltd. (Ansan, Republic of Korea). Deionized water was used in all experiments.

### 2.2. Preparation of Pristine CasNa, CasNa/GY, and CasNa/SO Coating Solution and Their Coated Papers

Pristine CasNa-, CasNa/GY-, and CasNa/SO-coated papers were prepared according to the compositions listed in Table 1. First, 10 g of CasNa was dissolved in 90 mL of deionized water by stirring at 600 rpm at 60 °C for 1 h. GY and SO were added to CasNa with stirring for 30 min. The prepared CasNa-based coating solutions were stored in a refrigerator at 3 °C for 24 h. The bar coating and double-layer techniques were employed to coat the CasNa-based solutions on the base paper. To create a smooth surface on the paper with an irregular surface, each coating solution was first coated onto base paper using a mayer bar (bar number: 30), and the coated papers were dried using a blow dryer at 110 °C for 1 min. Finally, double-layered pristine CasNa-, CasNa/GY-, and CasNa/SO-coated papers were prepared by coating the single-layered paper, and all of the coated papers were stored at 40 °C for 24 h. 

### 2.3. Chemical and Morphological Characterization

#### 2.3.1. pH, Viscosity, and Grammage

The pH values of the pristine CasNa, CasNa/GY, and CasNa/SO coating solutions were determined using a pH meter (F-17 LAQUA, Horiba Scientific Ltd., Kyoto, Japan). The viscosities of the solutions were determined using a Brookfield DVE viscometer (LV, AMETEK Inc., Berwyn, IL, USA) and a spindle number of 61. The spindle was rotated at 100 rpm. The grammage of the pristine CasNa-, CasNa/GY-, and CasNa/SO-coated papers was determined gravimetrically using a microbalance (Sartorius GmbH, Goettingen, Germany). 

#### 2.3.2. Morphological Structure 

The top surfaces of the base paper, pristine CasNa-, CasNa/GY-, and CasNa/SO-coated papers were observed using an Inspect F scanning electron microscope (SEM; FEI Co., Ltd., Hillsboro, OR, USA).

#### 2.3.3. Chemical Structure

The chemical structure of the GY, SO, pristine CasNa-, CasNa/GY-, and CasNa/SO-coated papers were recorded on a Spectrum 65 FTIR spectrometer (Perkin Elmer Co., Ltd., Waltham, MA, USA), with a spectral frequency range of 4000–500 cm^−1^ and 16 scans using the attenuated total reflection (ATR) mode.

### 2.4. Mechanical Properties

The burst strengths of the base paper, pristine CasNa-, CasNa/GY-, and CasNa/SO-coated papers were evaluated using the L&W SE 180 burst test machine (Lorentzen and Wettre Ltd., Kista, Sweden), according to KS M ISO 2785 [14]. The tensile strength and elongation at the breaks of the base paper, pristine CasNa-, CasNa/GY-, and CasNa/SO-coated papers were measured using an Instron 3367 universal test machine (UTM, Norwood, MA, USA), based on the KS M ISO 1924-2 standard protocols [15]. The load cell was 250 N, and the tensile velocity was set at 20 mm/min. All of the specimens were prepared in a rectangular shape, with 0.015 m width and 0.18 m length. 

### 2.5. Air Permeability

The air permeability of the base paper, pristine CasNa-, CasNa/GY-, and CasNa/SO-coated papers was measured using the Gurley air permeability tester (ABB Lorentzen & Wettre, Kista, Stockholm, Sweden) in accordance with KS M ISO 5636-3:2013. The permeation time was measured for 100 cc of air to permeate into the coated papers [16].

### 2.6. Surface Properties

The surface properties of the base paper, pristine CasNa-, CasNa/GY-, and CasNa/SO-coated papers were evaluated using Phoenix-MT contact angle measurements (SEO Co., Ltd., Suwon, Gyeonggi-do, Republic of Korea). The surface free energies (γ*^s^*) of the base paper and coated papers were calculated via the Owens–Wendt model, following the adhesion theory between solid and liquid [17]. Water with polar (γ*^s^*  =  72.8 mJ/m^2^, γ*^P^*  =  50.3 mJ/m^2^, γ*^D^*  =  22.5 mJ/m^2^) and diiodomethane with nonpolar (γ*^s^*  =  50.8 mJ/m^2^, γ*^P^*  =  50.4 mJ/m^2^, γ*^D^*  =  0.4 mJ/m^2^) characteristics were used as liquids for the analysis. 

### 2.7. Thermal Properties

To determine the thermal stability of the base paper, GY-, SO-, CasNa/GY-, and CasNa/SO-coated papers, thermogravimetric analysis (TGA) was performed with a 4000 TGA analyzer (PerkinElmer Co., Ltd., Waltham, MA, USA) at a heating rate of 10 °C/min and between 50 to 700 °C under N_2_ atmosphere, with a flow rate of 90 mL/min.

### 2.8. Statistical Analysis

Analysis of variance (ANOVA) was performed using IBM SPSS version 25 (SPSS Inc., Chicago, IL, USA). Duncan’s multiple range test was used to compare the differences between means. The significance level was set at *p* < 0.05.

## 3. Results and Discussion

### 3.1. CasNa Coating Solutions pH and Viscosity, and Coated Papers Grammage 

To investigate the effects of GY and SO on the pH and viscosity of the CasNa coating solutions, pH and viscosity measurements were conducted, as described in Table 1. The pH of the pristine CasNa solution was 6.6. The CasNa/GY and Cas/SO coating solutions did not significantly change the pH depending on the GY and SO content, respectively. It is reported that CasNa easily formed a gel near the isoelectric point [18]. To maintain the flowability of the CasNa coating solutions in commercial coating processes, such as gravure and comma, their pH should be maintained above the isoelectric point. The viscosity of the pristine CasNa solution was 58.1 mPa∙s. The viscosities of the CasNa/GY and CasNa/SO solutions decreased as the GY and SO contents increased. Siew et al. and Barreto et al., also reported a decrease in the viscosity of CasNa solution with increasing amounts of GY and SO [13,19]. This is explained by the decrease in the hydrodynamic volume of the components of CasNa solutions and the disruption of the protein-protein (e.g., hydrogen bond, ionic bond, and disulfide bond) and protein-solvent interactions by forming hydrogen bonds between CasNa and the plasticizer [13,19].

The control of the grammage in coated paper is crucial because it affects the physical properties, such as the gas barrier, and the mechanical properties. Therefore, grammages of pristine CasNa-, CasNa/GY-, and CasNa/SO-coated papers were prepared within the range of 90.1 to 94.3 g/m^2^. 

### 3.2. Coated Papers Morphologies

**Figure 1:**
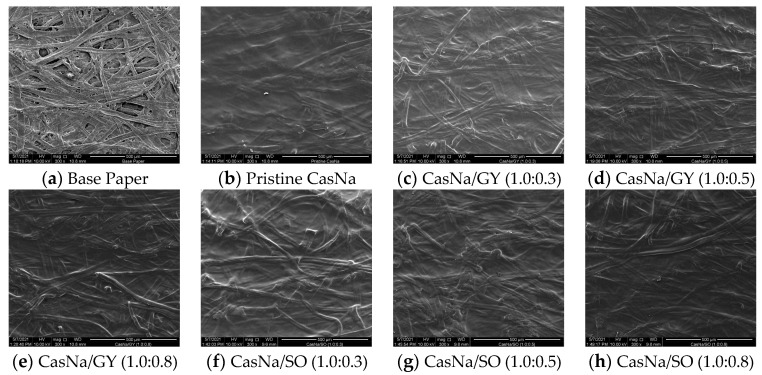
SEM images of (**a**) base paper, (**b**) pristine CasNa-coated paper, (**c**–**e**) CasNa/GY–coated papers and (**f**–**h**) CasNa/SO–coated papers (×300 magnification).

In the case of coated papers, a coating solution exists inside and on the top porous surface of the paper, which forms chemical and physical interactions between the coating solution and paper. The morphological structure of the coated papers affects their thermal and mechanical properties and air permeability. In this study, the effects of GY and SO on the morphological structure of CasNa/GY- and CasNa/SO-coated papers were observed using SEM, as shown in Figure 1. The base paper not coated with CasNa showed a porous structure composed of interwoven cellulose fiber (Figure 1a). The pores on the top surface of the pristine CasNa-coated paper were well covered by the pristine CasNa coating solution, although a portion of the fibers was still identified (Figure 1b). In addition, all CasNa/GY and CasNa/SO coating solutions penetrated the fibers well and covered the top surface of the paper without cracks (Figure 1c–h). This is related to the high compatibility and wettability between each coating solutions, with or without GY and SO, and the cellulose fiber, which may result in the penetration and adherence of all coating solutions to the base paper cellulose fibers [5,20]. GY and SO with low molecular weight diffuse into the CasNa matrix and facilitate mobility, in turn reducing the internal resistance of protein matrix. In addition, it is assumed that plasticized CasNa/GY and CasNa/SO coating layers are formed in the protein-plasticizer interactions with secondary force by disrupting the protein-protein interactions. These enhance the film forming process and flexibility by increasing the free volume and motion of the protein chains [21,22]. 

As a result, all of the coated papers tested here would help improve the mechanical properties and air permeability owing to morphological changes. This result is in agreement with those reported by other researchers [5,20].

### 3.3. Coated Papers Chemical Properties

**Figure 2:**
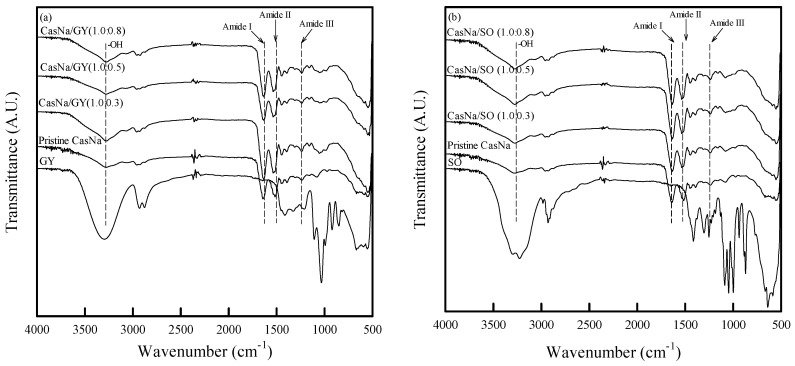
FTIR spectra of (**a**) CasNa/GY–coated papers and (**b**) CasNa/SO–coated papers.

The chemical interactions between the coating layer and cellulose are important to understand the changes in the chemical properties of coated papers depending on the plasticizer. The FTIR spectra of the CasNa/GY- and CasNa/SO-coated papers are shown in Figure 2. The CasNa/GY paper exhibited several characteristic peaks: stretching of -OH at 3293 cm^−1^, stretching of CH at 2931 and 2831 cm^−1^, bending of C-OH at 1415 cm^−1^, stretching of CO at 1033 cm^−1^, and vibration of C-C and C-O between 800–1150 cm^−1^ (Figure 2a) [23,24]. According to the FTIR spectra obtained for the CasNa/GY-coated papers, characteristic peaks between 1100–918 cm^−1^ appeared with the increasing GY content. In addition, the wavelengths of amide I, amide II, and amide III shifted from 1644 to 1633 cm^−1^, 1515 to 1519 cm^−1^, and 1236 to 1238 cm^−1^, respectively. These results are consistent with previous research showing that the characteristic peak of amide II in the extruded casein sheet was shifted by the addition of GY [23,25]. The CasNa/SO paper also exhibited several characteristic peaks: stretching of -OH at 3295 cm^−1^, stretching of CH at 2929 cm^−1^, stretching of C-OH at 1047 and 1087 cm^−1^, and bending of -OH at 1415 and 885 cm^−1^ (Figure 2b) [26,27]. The characteristic peaks between 1130 and 960 cm^−1^ of the CasNa/SO-coated paper gradually increased with the increase in the SO content. In addition, the wavelengths of amide I, amide II, and amide III shifted slightly from 1644 to 1633 cm^−1^, 1515 to 1517, and 1236 to 1238 cm^−1^, respectively. The pristine CasNa-coated paper also exhibited several characteristic peaks: stretching of -OH at 3276 cm^−1^, stretching of amide I (C=O) at 1644 cm^−1^, bending of amide II (NH) at 1515 cm^−1^, and deformation and bending of amide III (NH and C=O) at 1236 cm^−1^ (Figure 2) [10,23,28]. These results demonstrated that molecular interactions, such as hydrogen bonding between CasNa and plasticizers, and conformational rearrangement in CasNa occurred after GY or SO addition. 

### 3.4. Coated Papers Mechanical Properties

**Figure 3:**
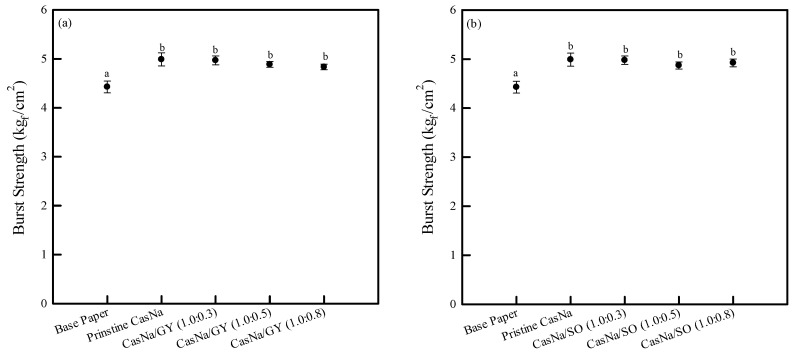
Burst strength of (**a**) CasNa/GY–coated papers and (**b**) CasNa/SO–coated papers. Lowercase letters (a,b) indicate statistically significant differences (*p* < 0.05).

**Figure 4:**
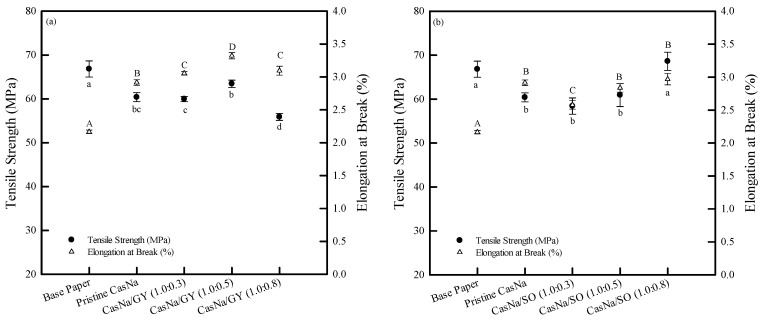
Tensile strength and elongation at break of (**a**) CasNa/GY–coated and (**b**) CasNa/SO–coated papers. Lowercase (a–d) and uppercase letters (A–D) indicate statistically significant differences of tensile strength and elongation at break, respectively (*p* < 0.05).

Packaging materials are exposed to various forces and pressures in the food and packaging processes, distribution, and storage environments. Therefore, the mechanical properties of CasNa/GY- and CasNa/SO-coated papers could correlate with the expected packaging material integrity under divergent forces and pressures in the packaging environment [10]. The mechanical properties of CasNa/GY- and CasNa/SO-coated papers were investigated as a function of the plasticizer content and compared to the base paper and pristine CasNa-coated paper, as shown in Figure 3 and Figure 4.

The base paper exhibited a burst strength of 4.4 kg_f_/cm^2^ (Figure 3). The pristine CasNa coating considerably improved the burst strength of the paper (5.0 kg_f_/cm^2^; Figure 3). This result is due to the penetration of CasNa into the pores and surface of the paper, which formed a smooth and continuous coating layer. However, the burst strength did not change significantly with the increase in the plasticizers GY and SO (Figure 3a,b). 

The base paper showed a tensile strength of 67.2 ± 2.3 MPa and an elongation at break of 2.4 ± 0.08% (Figure 4). The pristine CasNa-coated paper showed a tensile strength of 66.8 ± 1.8 MPa and an elongation at break of 2.2 ± 0.02%. This was because the presence of CasNa on the surface and pores between the cellulose fibers of the base paper negatively affected the hydrogen bonding between the cellulose fibers. The increase in the GY content in CasNa decreased the tensile strength, while it increased the elongation at break (Figure 4a). This result is consistent with previous studies by Chevalier et al. and Belyamani et al. [23,29]. CasNa/GY-coated paper can be interpreted as comprising CasNa, GY, and cellulose. If the interfacial interaction between CasNa/GY and cellulose is stronger than that between the cellulose fibers, then the tensile strength and elongation at break should increase. However, the interfacial interaction between CasNa/GY and cellulose appeared to be weaker than that between the cellulose fibers. In addition, GY was previously shown to reduce the intermolecular interactions between the CasNa molecules and increase the free volume, which facilitates the mobility of CasNa [29,30]. Similarly, the elongation at break of the CasNa/SO-coated papers increased with the increasing SO content (Figure 4b). However, the tensile strength was increased after 50% SO was added to the CasNa. The slight difference between the CasNa/GY- and CasNa/SO-coated papers might be due to differences in their properties, such as their molecular weight and the state of the plasticizers. GY (92 g/mol) is a viscous liquid, and SO (182 g/mol) is a solid powder. Thus, it is difficult for SO to penetrate the CasNa matrix compared to GY. Therefore, one could assume that some SO penetrated CasNa, acting as a plasticizer, while the other SO portion formed the crystalline region in the CasNa matrix. Consequently, CasNa/SO (1.0:0.5)- and CasNa/SO (1.0:0.8)-coated papers exhibited more rigidity than the pristine CasNa- and CasNa/SO (1.0:0.3)-coated papers [30].

### 3.5. Coated Papers Air Permeability

**Figure 5:**
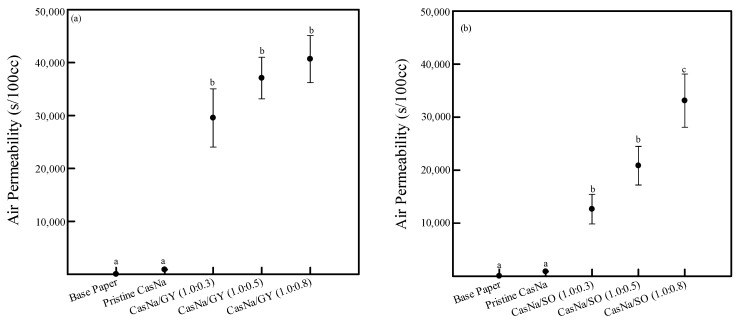
Air permeability of (**a**) CasNa/GY–coated papers and (**b**) CasNa/SO–coated papers. Lowercase letters (a–c) indicate statistically significant differences (*p* < 0.05).

Gas barrier properties are important for extending the shelf life and preserving the quality of various foods. In general, packaging materials must minimize the gases, including oxygen and water vapor, permeating the packaging [8,31]. Unfortunately, the paper’s porous structure renders it a poor gas barrier, which is a drawback that limits its application. In this study, air barriers were evaluated to investigate the effect of pristine CasNa, CasNa/GY, and CasNa/SO coatings on paper (Figure 5). The air permeability of the base paper was 17.0 s/100 cc due to its porous structure. After coating the paper with pristine CasNa, it reached 869.7 s/100 cc because the porous structure of the paper was filled and covered with a pristine coating solution (Figure 5). Furthermore, by raising the plasticizer concentration in the CasNa solution, the air permeability of the CasNa/GY- and CasNa/SO-coated papers also increased from 869 to 40,666 s/100 cc and 869 to 33,103 s/100 cc, respectively (Figure 5a,b). GY is a relatively hydrophilic structure compared to SO. Therefore, it seemed that hydrogen bonds were strongly formed between CasNa and GY compared to between CasNa and SO, which made air permeation difficult in the CasNa/GY-coated papers. As shown in the FTIR analysis (Section 3.3), GY and SO may reduce the strong interfacial interaction between CasNa molecules, positively affecting the formation of smooth films. In addition, as seen in the SEM analysis (Section 3.2), the paper pores were filled with CasNa/GY and CasNa/SO coating solutions without brittleness, which improved the air barrier of the paper. Faust et al., reported that the oxygen barrier of glycerol-containing pea-protein isolated film was improved with an increase in the glycerol concentration [22]. They reported that adding glycerol improves the flexibility and workability of pea-protein isolated film, which positively affects the oxygen barrier property. 

### 3.6. Coated Papers Surface Properties

To investigate the surface properties of the base paper and the pristine CasNa-, CasNa/GY-, and CasNa/SO-coated papers, their static contact angles were measured using water and diiodomethane. The static contact angles of the liquids strongly depend on the changes in the chemical and morphological structures of the CasNa-based coating layer, which depends on the plasticizer. The water and diiodomethane static contact angles for the base paper were 73.9° ± 0.62° and 31.0° ± 0.35°, respectively (Table 2). Notably, the pristine CasNa-coated paper showed decreases in the water and diiodomethane contact angles (61.2 ± 0.37° and 30.0 ± 0.87°; Table 2), suggesting that the hydrophilicity of the pristine CasNa-coated paper was enhanced. With the increasing GY or SO content in the CasNa, the polar surface energy changed from 11.2 to 12.5 mN and from 11.2 to 9.9 mN. The dispersive surface energy is associated with non-polarity and surface roughness. The dispersive surface energy increased slightly from 40.5 to 42.8 and from 40.5 to 43.8 mN with GY and SO, respectively (Table 2). Finally, the total surface free energies of the CasNa/GY- and CasNa/SO-coated papers were slightly higher than that of the CasNa-coated paper. Faust et al., reported that the addition of SO increased the surface energy of the polar parts in pea protein-isolated films [22]. However, in this study, the surface energy of the dispersive parts of the CasNa/GY- and CasNa/SO-coated papers increased more than the surface energy of the polar parts. This increase appears to be related to the morphology of the coated layers, as shown in Figure 1. Although the coating solutions covered the surface and pores of the fibers, the thickness of the coating layer remained uneven, owing to the morphological structure of the top surface of the base paper. To improve the physical properties of the coated paper, the coating solution must sufficiently penetrate the pores and completely spread on the paper surface [32]. In addition, the surface energy of the base paper was lower than those of the pristine CasNa-, CasNa/GY-, and CasNa/SO-coated papers (Table 2). This surface energy difference is related to the chemical structures of pristine CasNa, CasNa/GY, and CasNa/SO coating solutions, which may also affect the physical properties of the coated paper. Therefore, further studies are required on the wettability of the coated papers.

### 3.7. Coated Papers Thermal Stability

**Figure 6:**
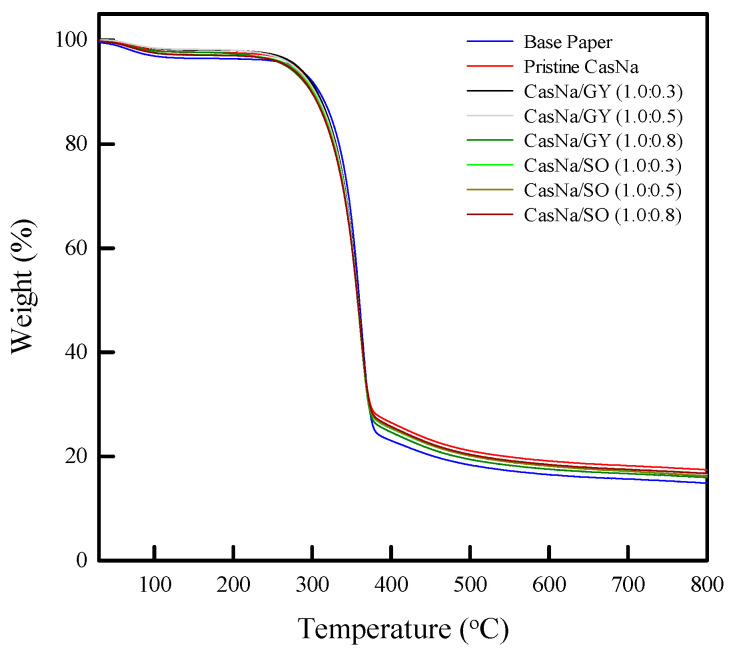
TGA curves of base paper, pristine CasNa–, CasNa/GY– and CasNa/SO–coated papers.

The base paper, pristine CasNa, CasNa/GY, and CasNa/SO-coated papers exhibited a two-step degradation process (Figure 6). The first degradation stage, at approximately 80 °C and 130 °C, was due to water evaporation. The second degradation stage, at around 230 °C and 260 °C, was due to the decomposition of the CasNa, plasticizer, and paper. The pristine CasNa-coated paper showed a 1 wt% higher decomposition temperature (70.2 °C) than that of the base paper (50.6 °C). After changing the GY content, the 1 wt% decomposition temperature increased from 70.2 °C to 77.6 °C in the CasNa/GY (1.0:0.5)-coated paper and then decreased with the rise in the GY concentration. Similarly, the pristine CasNa-coated paper also showed a higher 5 wt% decomposition temperature (278.6 °C) than the base paper (275.9 °C). With the increase in GY in CasNa, the 5 wt% decomposition temperature increased from 278.6 °C to 280.8 °C in CasNa/GY (1.0:0.3) and decreased to 266.9 °C in CasNa/GY (1.0:0.8). However, the 1 wt% and 5 wt% decomposition temperatures of the CasNa/SO-coated papers decreased with the increasing SO content. 

Such a change in the thermal stability of the CasNa-, CasNa/GY-, and CasNa/SO-coated papers can be attributed to the changes originating from the interfacial interactions between CasNa, plasticizers, and paper [28,33]. Overall, the CasNa/GY- and CasNa/SO-coated papers showed lower decomposition temperatures than the pristine CasNa-coated paper. The decrease in the thermal stability of the coated papers can be explained by the effects of GY and SO on the protein-protein interaction in CasNa [28]. Tarique et al., reported that arrowroot starch films prepared using glycerol showed a three-step thermal degradation pattern. The first step in the thermal decomposition of the films was related to the loss of water from the films. The second step of thermal decomposition happened to vary in the range of approximately 125–290 °C, which was related to the loss of the glycerol compound [34]. Bareeto et al., reported that the initial decomposition temperature of milk proteins decreased, which was associated with sorbitol affecting the protein-protein interactions [33].

## 4. Conclusions

In this study, coating solutions of pristine CasNa, CasNa/GY, and CasNa/SO with different plasticizer concentrations were prepared via solution blending and coated on the surface of the base paper. SEM confirmed that the pristine CasNa-, CasNa/GY-, and CasNa/SO-coated papers were well prepared, without any cracks on the surface. Compared to the base paper and pristine CasNa-coated paper, the air barrier properties of CasNa/GY and CasNa/SO were significantly improved as the GY and SO contents increased. The CasNa/GY-coated papers exhibited higher flexibility and air barriers than CasNa/SO, although the CasNa/SO-coated papers showed lower decomposition temperatures than all the other tested papers. Furthermore, the increase in both the plasticizer contents in the CasNa solution led to a rise in the polar surface more than the dispersive surface energy. The slight difference between the CasNa/GY- and CasNa/SO-coated papers might be related to changes in the chemical and morphological structures originating from the different molecular weights and states of the plasticizers. 

## Figures and Tables

**Table 1:** Compositions, pH, viscosity, and grammage of pristine paper, pristine CasNa–, CasNa/GY– and CasNa/SO–coated papers. Lowercase (^a–c^) and uppercase letters (^A–D^) indicate statistically significant differences of CasNa/GY–based coatings and CasNa/SO–based coatings, respectively (*p* < 0.05).

Sample Code	Compositions (g)				pH	Viscosity (mPa·s)	Grammage (g/m^2^)
	CasNa	D.I. Water	GY	SO			
Base Paper	-	-	-	-	-	-	80.0
Pristine CasNa	10.0	90.0	0.0	-	6.6	58.1 ^a,A^	90.1 ^a,A^
CasNa/GY (1.0:0.3)	10.0	90.0	3.0	-	6.7	50.8 ^b^	90.9 ^ab^
CasNa/GY (1.0:0.5)	10.0	90.0	5.0	-	6.7	45.7 ^c^	91.3 ^ab^
CasNa/GY (1.0:0.8)	10.0	90.0	8.0	-	6.9	45.7 ^c^	93.1 ^b^
CasNa/SO (1.0:0.3)	10.0	90.0	-	3.0	6.8	57.5 ^B^	91.1 ^AB^
CasNa/SO (1.0:0.5)	10.0	90.0	-	5.0	6.9	56.1 ^C^	92.2 ^B^
CasNa/SO (1.0:0.8)	10.0	90.0	-	8.0	6.9	51.7 ^D^	94.3 ^C^

**Table 2:** Contact angles and surface energy of (^a^) CasNa/GY–coated papers and (^b^) CasNa/SO–coated papers. Lowercase (^a–c^) and uppercase letters (^A–D^) indicate statistically significant differences of CasNa/GY–coated papers and CasNa/SO–coated papers, respectively (*p* < 0.05).

Sample Code	Static Contact Angle (°)		Surface Energy (mN/m^2^)		
	Water	Diiodomethane	Polar	Dispersive	Total
Base Paper	73.9 ± 0.62 ^aA^	31.0 ± 0.35 ^a^	5.1	41.3	46.4
Pristine CasNa	61.2 ± 0.37 ^bB^	30.0 ± 0.87 ^bBC^	11.2	40.5	51.7
CasNa/GY (1.0:0.3)	61.2 ± 0.47 ^b^	23.5 ± 0.59 ^b^	10.4	42.9	53.3
CasNa/GY (1.0:0.5)	60.5 ± 0.30 ^b^	23.5 ± 0.47 ^b^	10.7	42.9	53.6
CasNa/GY (1.0:0.8)	57.3 ± 0.04 ^c^	22.9 ± 0.36 ^b^	12.5	42.8	55.3
CasNa/SO (1.0:0.3)	61.9 ± 0.22 ^BC^	25.6 ± 0.35 ^C^	10.3	42.3	52.5
CasNa/SO (1.0:0.5)	62.5 ± 0.09 ^C^	22.9 ± 0.54 ^B^	9.6	43.3	52.9
CasNa/SO (1.0:0.8)	61.5 ± 0.22 ^B^	20.9 ± 0.16 ^D^	9.9	43.8	53.7

## Data Availability

The data presented in this study are available on request from the corresponding author.
